# The 90% effective dose of remimazolam in combination with sufentanil for sedation in patients undergoing gastroscopy

**DOI:** 10.3389/fmed.2026.1929896

**Published:** 2026-09-23

**Authors:** Yan Chen, Fan Jin, Wenjie Wang, Hongwei Wang

**Affiliations:** 1 Department of Anesthesiology, Zhuji People’s Hospital of Zhejiang Province, Zhuji, China; 2 Wuhan University of Science and Technology, Wuhan, China; 3 Department of Anesthesiology, Tongde Hospital of Zhejiang Province, Hangzhou, China

**Keywords:** biased-coin design, ED90, effective dose, gastroscopy, isotonic regression, remimazolam

## Abstract

**Objective:**

To determine the 90% effective dose (ED_90_) of remimazolam in combination with sufentanil for sedation in patients undergoing gastroscopy.

**Methods:**

Fifty-six patients aged 18–65 years with ASA physical status I–II and a BMI of 18–30 kg/m^2^ who were scheduled for gastroscopy were enrolled. All patients received 5 μg of sufentanil, followed 3 min later by an initial dose of 5 mg remimazolam. The dose was determined using a two-stage biased-coin design up-and-down sequential method, with each patient's dose based on the response of the preceding patient. Dose adjustments were made in 2.5 mg increments. For failure of sedation (negative response: coughing, body movement, or MOAA/S > 1 during pharyngeal passage), the dose for the next patient was increased deterministically by 2.5 mg; for success of sedation (positive response: MOAA/S ≤ 1), the dose was decreased with 11% probability or kept unchanged with 89% probability. ED_90_ and its 95% confidence interval (CI) were estimated using isotonic regression with bootstrap resampling. The incidence of adverse events during sedation was recorded.

**Results:**

The ED_90_ of remimazolam combined with 5 μg of sufentanil was 17.625 mg (95% CI: 15.467–20.000 mg). Six patients experienced coughing, and seven developed transient hypotension during sedation. No other adverse events were observed.

**Conclusion:**

The ED_90_ of remimazolam combined with sufentanil for gastroscopy sedation was 17.625 mg, with a low incidence of adverse events. These results provided a clinically relevant dosing benchmark for this promising drug combination.

## Background

Gastroscopy, a standard diagnostic and therapeutic procedure for upper gastrointestinal diseases, is increasingly performed as demand for digestive disease screening rises (1). However, the procedure can induce significant patient discomfort, including anxiety, pain, and vasovagal reactions. Consequently, moderate-to-deep sedation has become integral to contemporary practice, aiming to enhance patient comfort and procedural efficiency (2).

While propofol remains the primary sedative used in endoscopy, its narrow therapeutic index and associated cardiorespiratory risks, such as hypotension and airway compromise, remain clinical concerns (3–5). This has spurred interest in safer alternatives. Remimazolam, a novel ultra-short-acting benzodiazepine with a high affinity for GABA_a_ receptors, offers a favorable profile characterized by rapid onset, organ-independent metabolism, and minimal hemodynamic impact, making it a promising agent for procedural sedation (6). Although clinical studies have confirmed its non-inferior efficacy and potentially superior safety compared to propofol (7, 8), a critical knowledge gap persists regarding its optimal dosing.

Extant pharmacodynamic studies of remimazolam in gastroscopy have predominantly focused on estimating the 50% effective dose (ED_50_) using traditional sequential methods. However, the ED_50_ has limited clinical utility, as it ensures adequate sedation in only half of the population (9). Extrapolation from the ED_50_ to the clinically more relevant ED_95_ using conventional Dixon up-and-down methodology is statistically unreliable (10). To address this methodological and clinical gap, a more robust approach is required to directly estimate a higher quantile dose, such as the ED_90_, which is more closely aligned with clinical requirements and provides a more reliable basis for estimating the ED_95_.

This study therefore employed a methodologically novel design that combined the biased-coin design (BCD) with isotonic regression (via the Pool-Adjacent-Violators Algorithm, PAVA). The BCD, unlike deterministic up-down methods, incorporated a probabilistic element (e.g., an 11% probability of increasing the dose after failure and an 89% probability of repeating the same dose), allowing for efficient and stable exploration of the dose-response curve around the target quantile (11). Subsequently, the PAVA algorithm was applied to the collected binary response data to provide a non-parametric, monotonic estimate of the entire dose-response relationship, from which the ED_90_ and its confidence interval can be directly and reliably estimated (12).

Thus, the primary objective of this study was to directly and accurately determine the ED_90_ of remimazolam, in combination with a fixed dose of sufentanil, for achieving satisfactory sedation in patients undergoing gastroscopy, utilizing this advanced BCD-PAVA framework.

## Material and methods

### Study design and participants

This study was approved by the Ethics Committee of the Zhuji People's Hospital in Zhejiang Province (Approval No.: 2022kelun0613). The trial was registered at the Chinese Clinical Trial Registry (CHICTR2200062381). Patients who planned to undergo gastroscopy under sedation in the Gastrointestinal Suite of Zhuji People's Hospital from August to September 2022 were selected after obtaining written informed consent.

Patients eligible for enrollment in this study were individuals aged 18–65 years, with American Society of Anesthesiologists Physical Status (ASA PS) of I and II, and a body mass index (BMI) between 18 and 30 kg/m^2^. Exclusion criteria were severe respiratory diseases (acute respiratory infection, obstructive sleep apnea syndrome, uncontrolled asthma, and acute exacerbation of chronic obstructive pulmonary disease), difficult airway (Mallampati class IV, limited mouth opening, and thyromental distance less than 6 cm), history of sleep apnea, acute heart failure, unstable angina pectoris, or myocardial infarction within 6 months, history of renal or hepatic dysfunction, hemodynamic instability (Mean arterial pressure, MAP <65 mmHg; systolic blood pressure, SBP >180 mmHg; or diastolic blood pressure, DBP >110 mmHg), allergy or contraindication to benzodiazepines (BZDs) or propofol, mental disorders (schizophrenia, mania, bipolar disorder, and other mental disorders) or cognitive impairment, long history of taking mental or sedative hypnotic drugs, history of drug or alcohol abuse [alcohol abuse: average daily consumption of more than 2 units of alcohol (1 unit = 360 mL beer or 45 mL 40% liquor or 150 mL wine)], pregnancy, lactation or plans for pregnancy within 3 months.

Withdrawal criteria were loss to follow-up, death from any cause, severe drug allergy, severe respiratory depression, other complications requiring urgent treatment, or missing data.

### Sedation protocol and sedation depth assessment

All patients followed a regular fasting protocol before their gastroscopy procedures. Upon entering the procedure room, non-invasive blood pressure (NIBP), pulse oxygen saturation (SpO_2_), and electrocardiography (ECG) were established. Ringer's solution was administered at an infusion rate of about 15 mL/kg^−1^·h^−1^. Prior to induction, patients received simethicone (6 mL) and oxygen via a nasal cannula at a flow rate of 3–5 L/min. All patients received a fixed intravenous bolus of 5 μg sufentanil. The fixed-dose regimen for remimazolam was adopted based on population pharmacokinetic analyses suggesting that no clinically relevant dose adjustments are required for covariates including body weight (13), and this approach is also consistent with the dosing strategy used in the pivotal phase III trial. For sufentanil, the fixed 5 μg dose falls within the established 0.1 μg/kg ED_95_ range for our cohort (mean weight 62.9 kg) (14). Three minutes later, the initial dose of remimazolam was administered intravenously.

The sedation depth was assessed at the critical moment of the gastroscope passing through the pharynx, using the Modified Observer's Assessment of Alertness/Sedation (MOAA/S) scale. A positive response was strictly defined as MOAA/S ≤ 1 at this time point. A negative response was defined as MOAA/S > 1, signifying inadequate sedation, which could manifest as coughing, purposeful movement, verbal response to commands. The cutoff of MOAA/S ≤ 1 was chosen to ensure a sufficiently deep sedation level that reliably prevents body movement and gag reflex during scope passage, which is essential for procedural success and patient safety. MOAA/S scores of 1–2 are conventionally defined as deep sedation, and this threshold has been widely adopted in recent gastroscopy sedation studies (15).

Rescue Sedation: If the initial dose resulted in a negative response, supplemental boluses of remimazolam (2.5 mg each) could be administered at intervals of at least 1 min, allowing time for the drug to reach its peak effect. A maximum of two supplemental doses was permitted. If adequate sedation (MOAA/S ≤ 1) was still not achieved after these supplemental doses, propofol (20–50 mg boluses) was administered as rescue medication to complete the procedure, and this case was recorded as a negative response for the purpose of dose estimation.

### Dose-escalation strategy: two-stage biased-coin design (BCD)

The dose of the initial remimazolam bolus for each patient was determined sequentially using a two-stage BCD, targeting the 90% effective dose (ED_90_). The dose for any given patient depended on the response of the immediately preceding patient.

#### Stage 1 (initial escalation)

The first patient received a starting dose of 5.0 mg. Following a negative response, the dose for the next patient was increased by a fixed increment of 2.5 mg. This deterministic escalation continued until the first occurrence of a positive response. The dose administered to this patient marked the transition to Stage 2. This patient, while triggering the transition, was considered part of Stage 1 and received the dose that produced the positive response.

#### Stage 2 (BCD phase)

Starting from the dose level that first yielded a positive response, the adjustment rule incorporated a probabilistic element. If a patient had a negative response, the dose for the next patient was increased by 2.5 mg (deterministic rule). If a patient had a positive response, the dose for the next patient was determined through biased-coin randomization implemented in R, with the parameter *Γ* set at 0.9. This *Γ* value was chosen because ED_90_ is the target quantile; the formula b = (1-*Γ*)/*Γ* = 0.111 gives an 11% probability of decreasing the dose after a success, which optimizes the allocation of patients around the 90th percentile. This meant there was an 11% probability (b = 0.111) of decreasing the dose by 2.5 mg and an 89% probability (1-b = 0.89) of keeping the dose unchanged for the next patient. The subsequent patient in Stage 2 received the same dose level as the transitioning patient, and the probabilistic BCD adjustment rule applied from that point forward.

### Stopping rule and outcomes

The study continued in Stage 2 until a total of 45 patients with positive responses were enrolled, a sample size predetermined based on simulation studies to provide stable estimation of the ED_90_ (16). The stopping rule of 45 positive responses applies exclusively to Stage 2; patients enrolled in Stage 1 were not counted toward this target, as the sample size calculation was based on the BCD phase alone. Data from all enrolled patients in Stage 2 were included in the final analysis.

The rationale for this two-stage design is twofold. First, when the starting dose is substantially lower than the target ED_90_, a pure BCD from a far-below-target starting dose would require an excessive number of patients to gradually ascend to the target dose range (17). The deterministic escalation rule in Stage 1 serves to rapidly advance the dose from a conservative starting point to the vicinity of the target ED_90_, minimizing patient exposure to subtherapeutic doses (11). Second, transition to Stage 2 occurs immediately upon the first positive response, after which all subsequent dose allocations follow the BCD.

The main objective of this study was to determine the ED_90_ of remimazolam in combination with sufentanil for sedation during gastroscopy. Secondary outcomes were as follows (1): induction dose of remimazolam, induction time, procedural time, and awakening time. Induction time was defined as the duration from immediately after the injection of remimazolam to an MOAA/S score ≤1. The procedural time was defined as the duration from insertion to removal of the gastroscope. Awakening time was defined as the duration from the last injection of remimazolam to three consecutive MOAA/S scores ≥ 5 (2); MAP and HR at the following time points: arrival at the gastrointestinal suite (T_0_), 1 min after sufentanil injection (T_1_), 1 min after remimazolam injection (T_2_), gastroscope passing through the throat (T_3_), immediately after gastroscopy (T_4_), awakening time (T_5_) (3); adverse events, including injection pain, hypotension (MAP <80% of baseline), hypoxia (SpO_2_ < 90%), bucking (coughing), dizziness, nausea and vomiting, bradycardia (HR <60 bpm), and use of flumazenil.

### Statistical analysis

Statistical analysis was carried out using SPSS 23.0 (SPSS Inc., Chicago, IL, United States) and R software (version 3.5.3, https://www.R-project.org), while graphical representations were generated using GraphPad Prism 8.0. Normally distributed continuous variables were presented as mean and standard deviation (mean ± SD), whereas skewed continuous variables were expressed as median (M) and interquartile ranges (IQR). Categorical variables were presented as numbers and percentages.

The ED_90_ and its 95% confidence interval (CI) were estimated using the pooled-adjacent-violators algorithm (PAVA) for isotonic regression in combination with bootstrapping. Generalized estimating equations (GEE) were utilized to analyze mean arterial pressure (MAP) and heart rate (HR). Bonferroni correction was applied to adjust the p-values for each time point and each dose of remimazolam due to multiple comparisons. A *p*-value <0.05 was considered indicative of statistical significance.

## Results

### Patient enrollment and characteristics

A total of 60 patients were screened between August and September 2022. Four patients were excluded: one declined participation, one had incomplete data, and two required polypectomy during the procedure. The two polypectomy cases were excluded post-hoc because therapeutic procedures prolong the gastroscopy duration and may alter the sedation requirements beyond the scope of our study, which was designed for diagnostic procedures; however, we acknowledge that this exclusion is a limitation. Consequently, 56 patients were included in the final analysis. The cohort comprised an equal sex distribution (28 males, 28 females) with a median age of 49.5 years (IQR, 21.0 years). Detailed baseline characteristics are presented in Table 1.

**Table 1:** Baseline characteristics.

Variables	Total (*n* = 56)
Sex (male/female), *n* (%)	28 (50.0%)/28 (50.0%)
Age [yr, median (IQR)]	49.5 (21.0)
Height (m, mean ± SD)	1.7 ± 0.1
Weight (kg, mean ± SD)	62.9 ± 10.5
BMI (kg/m^2^, mean ± SD)	22.9 ± 2.7
ASA, *n* (%)	
I	48 (85.7%)
II	8 (14.3%)

Data are presented as mean ± SD or median (IQR) or number (%). BMI, body mass index; ASA, American Society of Anesthesiologists.

### Primary outcome: ED_90_ of remimazolam

During the second stage of the biased-coin design, the administered remimazolam doses among the 56 patients converged to seven discrete levels: *Ω* = {5.0, 7.5, 10.0, 12.5, 15.0, 17.5, 20.0} mg. Using isotonic regression via the pooled-adjacent-violators algorithm (PAVA) and bootstrapping, the estimated ED_90_ of remimazolam (in combination with 5 µg sufentanil) for successful gastroscopy sedation was 17.625 mg (95% CI: 15.467–20.000 mg).

Table 3 presents the observed and PAVA-adjusted response rates at each dose level. The PAVA adjustment enforces monotonicity in the dose-response relationship, ensuring that response rates do not decrease as dose increases. The adjusted rates provide the basis for the ED_90_ estimation.

### Secondary outcomes

#### Sedation efficacy

The mean induction dose of remimazolam was 15.4 mg (SD: 5.0 mg). A total of 45 patients (80.4%) achieved a positive response (MOAA/S ≤ 1), while 11 patients (19.6%) had a negative response. Propofol rescue was required for one patient among the negative responders (Figure 1).

**Figure 1:**
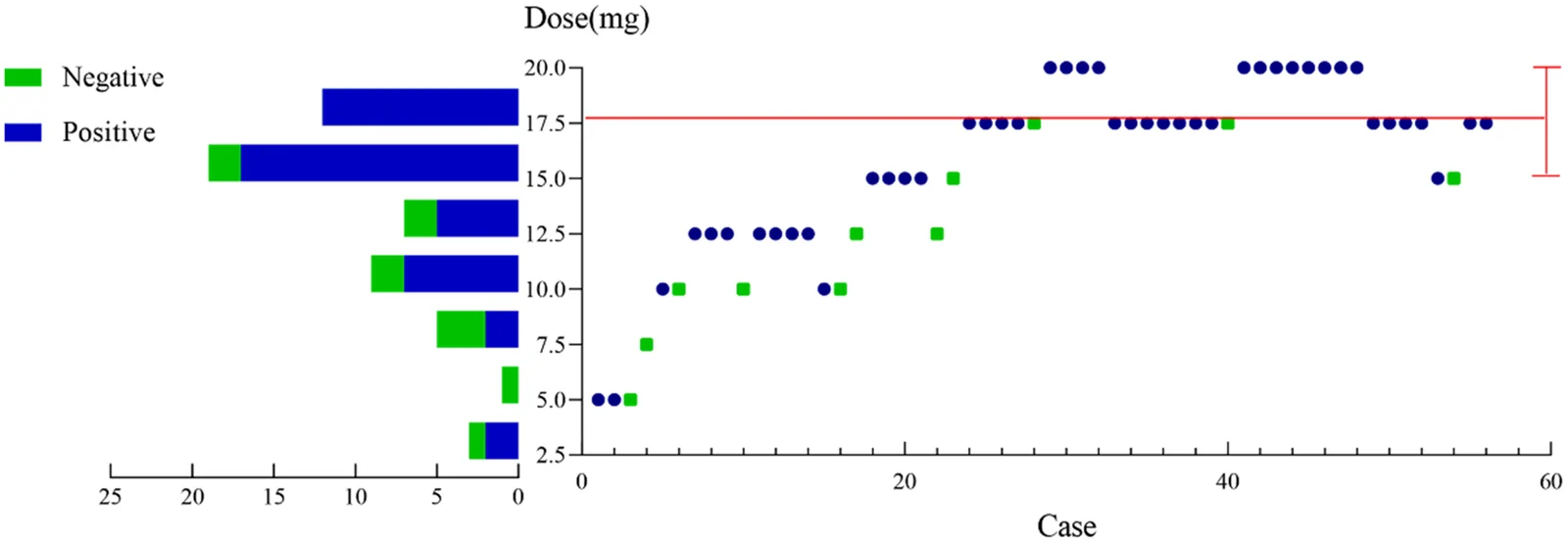
The right side of the ordinate shows the results of the complete BCD. (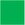) denotes negative cases, (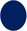) denotes positive cases, and the line (x) indicates the estimated value of ED90 and 95% CI. The left side of the ordinate shows that BCD aggregate the cases of dose near ED90 that can be effectively estimated in a small sample size.

#### Procedural timings

The median induction time was 24.0 s (IQR, 8.0 s), the median operation time was 5.3 min (IQR, 1.0 min), and the median awakening time was 8.0 min (IQR, 2.0 min) (Table 2).

**Table 2:** Secondary outcomes.

Variables	Total (*n* = 56)
Induction dose of remimazolam,（mg）	15.4 (5.0)
Induction time (s)	24.0 (8.0)
Operation time (min)	5.3 (1.0)
Awakening time (min)	8.0 (2.0)
Positive response, *n* (%)	45 (80.4%)
Negative response, *n* (%)	11 (19.6%)

Data are presented as median (IQR) or *n* (%).

**Table 3:** Observed and PAVA-adjusted response rates at each remimazolam dose level.

Dose (mg)	No. of Patients	Observed Positive Responses	Observed Response Rate (%)	PAVA-Adjusted Response Rate (%)
5.0	3	1	33.3	33.3
7.5	1	0	0	33.3
10.0	5	2	40.0	57.1
12.5	9	6	66.7	66.7
15.0	7	5	71.4	71.4
17.5	19	17	89.5	85.5
20.0	12	11	91.7	91.7

#### Hemodynamic effects

The numbers of patients in the seven dose groups were 3, 1, 5, 9, 7, 19, and 12, respectively. For the hemodynamic stability analysis, dose levels with fewer than 6 patients were excluded, focusing on the 12.5, 15.0, 17.5, and 20.0 mg groups. Generalized Estimating Equations (GEE) with Bonferroni correction were employed.

#### Mean arterial pressure (MAP)

No statistically significant difference in MAP was observed across dose levels after adjusting for time points (Wald *χ*^2^ = 1.524, *P* = 0.677). However, MAP varied significantly across time points after adjusting for dose levels (Wald *χ*^2^ = 117.572, *P* < 0.001). Specifically, MAP at baseline (T0) and post-induction (T1) were comparable (all *P* > 0.05), but both were significantly higher than MAP at all subsequent procedural time points (T2–T5, all *P* < 0.05) (Figure 2).

**Figure 2:**
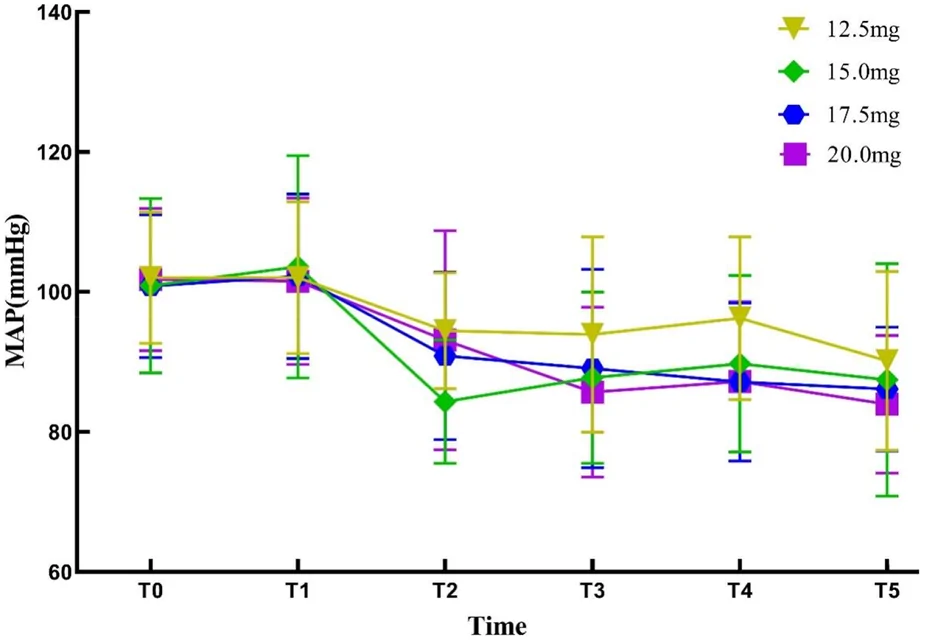
MAP at different time points and different dose levels.

#### Heart rate (HR)

No statistically significant difference in HR was found across dose levels after adjusting for time points (Wald *χ*^2^ = 0.781, *P* = 0.854). HR varied significantly across time points after adjusting for dose levels (Wald *χ*^2^ = 31.383, *P* < 0.001). *Post-hoc* analysis indicated that HR at T5 was significantly lower than at T2 (mean difference, −6.0 bpm; 95% CI: −11.0 to −1.1), T3 (mean difference: −4.5 bpm; 95% CI: −8.0 to −1.0), and T4 (mean difference: −3.2 bpm; 95% CI: −5.5 to −1.0) (Figure 3).

**Figure 3:**
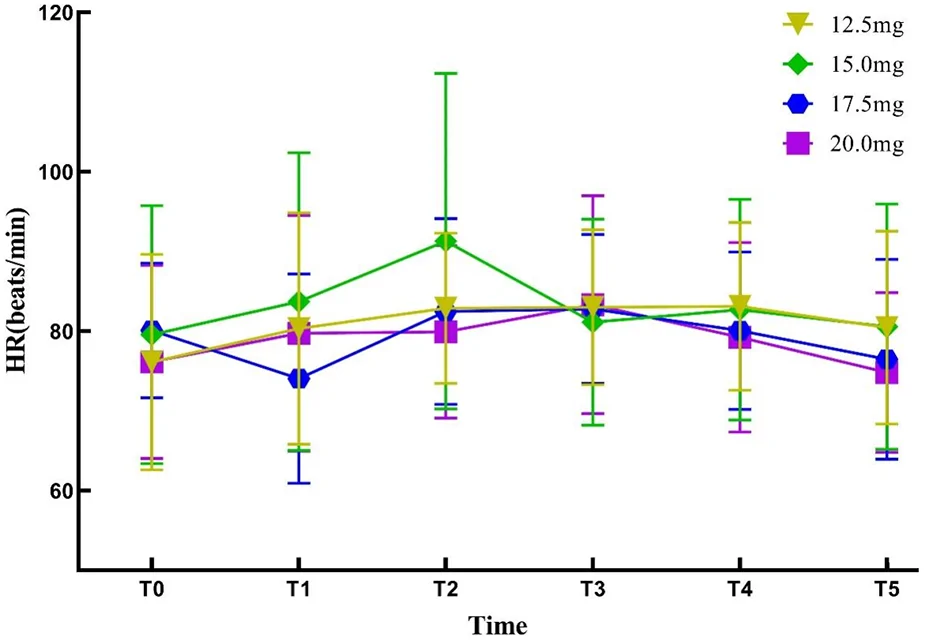
HR at different time points and different dose levels.

### Safety and adverse events

No instances of injection pain, bradycardia, hypoxia (SpO₂ < 90%), vertigo, nausea, or vomiting were reported. Six patients (10.7%) experienced coughing, and seven patients (12.5%) had transient hypotension during sedation, all of which resolved with standard interventions.

## Discussion

In this study, BCD and isotonic regression were used to determine the ED_90_ of remimazolam in combination with sufentanil for sedation during gastroscopy. Our findings revealed that the ED_90_ during sedation of gastroscopy was 17.625 mg (95% CI: 15.467–20.000 mg). Furthermore, Remimazolam had a relatively mild impact on circulatory parameters.

Remimazolam is widely used for endoscopic sedation, but alone it has low success and repeated dosing increases adverse events, so combination with opioids is preferred (18, 19). The starting dose of 5 mg and the incremental dose of 2.5 mg in this combination regimen were selected based on multiple lines of evidence: the approved product labeling for remimazolam in China recommends this regimen for gastroscopy sedation; a recent multicenter randomized controlled trial by Park et al. (20). also employed this 5 mg/2.5 mg regimen; and a dose-comparison RCT (21) demonstrated that higher initial doses (0.3–0.4 mg/kg), while achieving higher success rates, were associated with significantly increased adverse events including vertigo and prolonged recovery. Thus, the 5 mg starting dose offers a conservative balance between efficacy and safety, while the 2.5 mg increment allows safe and precise titration.

Sufentanil, a specific *μ* opioid receptor agonist, has been a popular choice during endoscopy due to its effective analgesic properties and mild impact on respiration and circulation. Previous studies have demonstrated a reduction in sedative dosage and fewer adverse events when sufentanil is used in combination to sedate the patient during endoscopy (22, 23). Zhu et al. (14) determined that the ED_95_ of sufentanil for endoscopy was 0.1 μg/kg (with a 95% confidence interval of 0.079–0.422 μg/kg). Given potential respiratory depression, asphyxia, and other adverse events associated with high doses of sufentanil, we selected a single dose of 5 μg in this study. Additionally, to synchronize the onset times of sufentanil and remimazolam and maximize their synergistic effects in sedation, sufentanil was administered 3 min prior to the intravenous injection of remimazolam.

Methodologically, this study represents a significant step forward. While traditional sequential or Dixon up-and-down methods were frequently used to estimate the ED_50_ of sedatives (23), the ED_50_ has limited clinical relevance as it ensures efficacy in only half the population. Moreover, these methods did not reliably estimate extreme quantiles of the dose-response curve (12). In contrast, the BCD with isotonic regression allowed for direct and accurate estimation of higher quantiles like the ED_90_, providing a dose that is both statistically robust and clinically applicable, a methodology increasingly validated in recent pharmacological studies (24–26). Our finding of an ED_90_ of 17.625 mg directly addressed the need for a practical dosing target that aimed to be effective for the vast majority of patients, thereby advancing beyond the conventional focus on median effective doses.

To place our findings in context, several recent ED_90_ studies of remimazolam have been published. For instance, Guo et al. (27) reported an ED_90_ of 0.300 mg/kg in elderly patients undergoing ERCP, and Tao et al. (28) found an ED_90_ of 0.28 mg/kg in pediatric neurosurgery. When converted to a weight-based dose using our mean body weight of 62.9 kg, our ED_90_ (17.625 mg) corresponds to approximately 0.28 mg/kg, which is remarkably consistent with Tao et al.'s (28) estimate. This similarity across different patient populations and procedures suggests a stable pharmacodynamic profile of remimazolam when combined with opioids. However, our fixed-dose approach may limit generalizability to patients at the extremes of body weight, and the relatively wide 95% CI (15.5–20.0 mg) indicates some residual uncertainty; clinicians should consider individual patient factors when applying this dose.

Regarding safety, our data indicated that the combination of remimazolam and sufentanil had a minimal and likely non—dose—dependent effect on circulation within the studied dose range. Although mean arterial pressure (MAP) at later procedural time points was significantly lower than at baseline, the absolute decrease was clinically modest (<20 mmHg), consistent with previous reports (29, 30). The adverse event profile was favorable, with no serious complications reported. Coughing during induction in six patients was likely related to initial inadequate sedation depth, and transient hypotension in seven patients resolved spontaneously. This overall pattern reinforces the favorable safety reputation of remimazolam, particularly when used in combination with a low-dose opioid.

The results have direct and practical implications for clinical practice. For the young to middle-aged, ASA I-II patient population studied, an induction dose of approximately 17.6 mg of remimazolam following 5 µg of sufentanil provided a high probability of successful sedation. Nevertheless, because the 95% CI ranges from 15.5 to 20.0 mg, clinicians should recognize that a dose near the lower bound may be insufficient for some patients, and titration to effect remains essential. This evidence-based benchmark could guide clinicians in achieving efficient procedural conditions while minimizing the risks associated with inadequate sedation or the need for excessive rescue dosing, thereby potentially improving both patient comfort and procedural workflow.

A limitation of our fixed-dose strategy is that body weight affects central distribution volume (31), so the 17.625 mg ED_90_ translates to ∼0.21–0.35 mg/kg across our BMI range (18–30 kg/m^2^). This may cause relative underdosing in heavier patients and overdosing in lighter ones. Thus, generalization to patients at weight extremes requires caution, and future weight-based dosing studies are warranted.

This study has some limitations. First, sedation was maintained to achieve MOAA/S ≤ 1, and objective monitoring tools such as the bispectral index were not employed to assess the level of sedation. This may have an impact on the dose of remimazolam administered. However, a previous study found a linear correlation between BIS and MOAA/S (32). Second, we only enrolled young and middle-aged patients, and whether remimazolam combined with sufentanil is suitable for all gastroscopy patients requires further investigation. Finally, although patient enrollment was completed in September 2022, the manuscript was delayed by subsequent methodological re-analysis and personnel changes within the research team. The pharmacological principles and clinical relevance of our findings remain unaffected by this time lag.

In conclusion, this study determined the ED_90_ of remimazolam when combined with sufentanil for gastroscopy sedation to be 17.625 mg, with a low incidence of adverse events. These findings provide a clinically relevant dosing benchmark for this promising drug combination.

## Data Availability

The raw data supporting the conclusions of this article will be made available by the authors, without undue reservation.
